# Editorial: Diagnostic Pathology continues to rise

**DOI:** 10.1186/1746-1596-3-48

**Published:** 2008-12-16

**Authors:** Klaus Kayser

**Affiliations:** 1 UICC-TPCC, Institute of Pathology, Charite, Berlin, Germany

## Editorial

Nearly three years have passed since the first issue of our journal Diagnostic Pathology had been accessible via our open access publisher BiomedCentral. About 150 scientific articles have been published since then. Therefore, we want to express our deep gratitude to all authors who submitted their articles. They took into account two constraints, namely paying a publication fee, and, in addition, knowing that our journal has not yet been included into the citation index list.

We are fully aware about the situation of our colleagues working in the scientific or health care environment: scientific publications in journals that are not listed in the citation index are considered of low quality. They usually are of no value for administrative or professional purposes. Therefore, we appreciate your support of our journal even more. In addition, you can be assured that we are trying hard to fulfil the citation index conditions. Unfortunately, we have to wait for a period of three years when applying for it. On the other hand, we do think that the citation index procedure is contradictory in itself: Why have newly established journals to wait for three years in our days? Do we not have several tools to measure all necessary numbers within a couple of seconds? Why a board of justices is mandatory for including or excluding new applicants when all necessary criteria can be found and graded by a simple Internet search?

We leave the answer of these (and similar) questions to our readers. On the other hand, we ask for your understanding that we are a little proud of the real data of our journal Diagnostic Pathology: at first the high and still increasing number of colleagues who read the articles published in our journal; secondly, the number of our published articles that are cited in other journals; and thirdly, the number of colleagues who have registered in our journal. These data stand for themselves: at average more than one thousand colleagues read an article published in Diagnostic Pathology within a period of six months after publication. This holds true for all kinds of articles, also for case reports. We derive from these data that our colleagues are really interested in case reports and, even more interesting, that case reports are a satisfactory source of medical information. Obviously it is attractive to demonstrate medical findings at an individual patient – oriented basis, which can be as instructive as that of a statistical environment.

**Figure 1:**
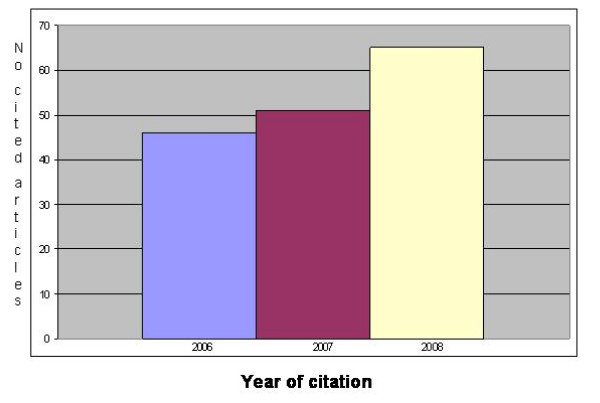
Number of articles cited in Journal of Diagnostic Pathology (<November 2008).

The number of articles cited in other scientific journals is, of course, dependent upon the time the journal does exist, and the period between the date of publication and that of citation. Unfortunately, we could not find published data about the relationship between the length of this period and the scientific impact. We assume that it will take at least six months until an article can be included in the reference list of another article, and that probably additional six months will pass until the citing article will be published, or printed. Our own data are depicted in figure 1 showing the citations of articles published in our journal Diagnostic Pathology in relation to the date of citation. A total of 162 articles have been cited until the end of November 2008.

As we have mentioned numerous colleagues still register in our journal Diagnostic Pathology with a nearly constant rate of about 30 new registrations per month. All in all we have more than 1000 registered colleagues until now. Everybody can read our journal without being registered; thus, we derive from these data that these colleagues probably read our journal regularly.

In addition to increase the scientific value of our journal we also want to encourage colleagues who are working in developing countries to submit or continue in submitting their articles to our journal. Although it is not in our power to release authors from the publication fee, we are doing our best to convince the publisher for providing adequate waivers.

As we have already discussed in our previous editorials, we are still engaged in establishing new tools for easier publication and more appropriate distribution of information via our journal Diagnostic Pathology. This will hopefully include the possibility of virtual slide integration as well as automated access to search machines such as that of the National Institute of Health. The discussion about these and other ideas with the publisher has started, and we do hope that they will be successful implemented in the near future.

From our point of view, a successful year is coming to its end. We acknowledge that not all aims could be reached; however, a big step forward could be done.

We wish all our authors, readers, reviewers, and our publication team a Merry Christmas and a Happy and Healthy New Year.

